# Editorial: Bioactive Phytochemicals in Asteraceae: Structure, Function, and Biological Activity

**DOI:** 10.3389/fpls.2019.01464

**Published:** 2019-11-14

**Authors:** Marina Soković, Helen Skaltsa, Isabel C. F. R. Ferreira

**Affiliations:** ^1^Mycological Laboratory, Department of Plant Physiology, Institute for Biological Research, Siniša Stanković, University of Belgrade, National Institute of Republic of Serbia, Belgrade, Serbia; ^2^Department of Pharmacognosy and Chemistry of Natural Products, School of Pharmacy, National and Kapodistrian University of Athens, Athens, Greece; ^3^Centro de Investigação de Montanha (CIMO), Instituto Politécnico de Bragança Campus Santa Apolónia, Bragança, Portugal

**Keywords:** bioactivity, secondary metabolites, Asteraceae (compositae), Arctium, Helichrysum, Baccharis, Cynara, Eremanthus

The ongoing era of natural matrices implies their importance in drug discovery, characterized with the isolation and identification of compounds, as well as assessment of their biological potential. Promising results obtained in recent years prompted scientific community to intensify this field of research, which resulted in numerous publications, patents, and ready-to-use drugs of natural origin. Furthermore, studies on numerous representatives of Asteraceae family revealed presence of chemically diverse natural products, with significant pharmaceutical and medicinal potential. With this in mind, the fortcoming issue was designed towards publication of articles (three research and two review) related to medicinal properties of plants belonging to Asteraceae family.

The first review article summarizes facts on chemical constituents and medicinal properties of *Arctium* species. As it will be elaborated througout the article, biologial potential of these species cannot be attributed strictly to the presence of a single compound, but could be rather linked to several major groups of compounds, such as: polysaccharides, terpenes/terpenoids, carboxylic and fatty acids, methoxypyrazines, phytosterols, and many others. As for the biological potential, several activities associated with major health issues have been demonstrated: antidiabetic, anticancer, antioxidant, hepatoprotective, gastroprotective, antimicrobial, antiallergic, and anti-inflammatory (Wang et al.).

The second review article covers the broad area of *Helichrysum areanrium* (L) Moench aka Sandy Everlasting biological potential. This perennial plant, indigenus to Europe and Asia, has long traditional use as a remedy for several ailments, though it mainly beneficially affects the liver (a protective and detoxifying agent). Most of the bioactives have been identified in the flowering part of *H. arenarium*: mainly flavonoids, but also phenolic acids, coumarines, pyrones, chalcones, sterols, glycosides, and lignans. Having in mind its pharmaceutical potential on one hand, and endangered status of the wild growing populations in several European countries (including Serbia) on the other, the species should be considered to be cultivated as a crop which would certainly enable continuous plant yield with uniform chemical characteristics (Pljevljakušić et al.).

As for the research articles, they cover the medicinal potential and underlying chemical background of three species, insufficiently known to research studies, but with great ethnobotanical legacy.

To start with, essential oil of *Baccharis dracunculifolia* DC, wild growing as a bush in Brazil and several other parts of South America exerted potential in several aspects of biological activities. Nevertheless, antimicrobial potential of the oil obtained from aerial plant parts has been poorly examined so far. Chemical analysis revealed presence of 30 compounds, with oxygenated sesquiterpenes spathulenol and trans-nerolidol as the main ones (27% and 23%, respectively). Regarding antibacterial potential, the essential oil obtained from aerial parts at flowering period exerted bacteriostatic/fungistatic and bactericidal/fungicidal activity towards several foodborne pathogens as well as clinically relevant pathogens including *Staphylococcus aureus*, *Pseudomonas aeruginosa*, *Aspergillus fumigatus*, etc. (Cazella et al.).

Another Asteraceae family member, Candeia or *Eremmanthus erythropappus* (DC) McLeisch native to Brazilian tropical savana region (cerrado), has long traditional use as a wound healer and analgetic. These specified medicinal properties, validated throughout the history of Brazilian inhabitants, have been attributed to fairly high content of sesquiterpene alcohol (-)-α-bisabolol. Bisabolol is very appreciated in cosmetic industry due to numerous skin beneficial activities, including antimicrobial, antiinflammatory, and wound healing effects. Additionaly, it smoothes skin surface making it soft and appealing. Due to over-exploitation a of Candeia timber to obtain sufficient amount of (-)-α-bisabolol, an effort has been made towards identifying a terpene synthase gene (EeBOS1) responsible for the production of this compound. The obtained results indicate that sustainable production of (-)-α-bisabolol without endangering natural populations seems possible (https://www.frontiersin.org/researchtopic/7042)Albertti et al.

In the last research study, conducted by Petropoulos et al., nutritional value and bioactives in cultivated *Cynara cardunculus* L. (Asteraceae) from Greece were characterized. Immature capitula of cardoon, suitable for human consumption, were used to obtain infromation on carbohydrate, protein, fat, and ash content, whereas different plant parts including seeds (used to obtain oils) were used to estimate free sugars, fatty and phenolic acids content, as well as antioxidant potential. Obtained results point to the fact that cardoon plant is rich in nutrients as well as bioactive molecules which may be widely used in pharmacy. Furthermore, due to the favorable chemical composition, oil obtained from the cardoon seeds may serve as an appropriate substitute for more commonly used vegetable oils, which may be applicable in food industry.

## Author Contributions

All authors contributed equally.

## Conflict of Interest

The authors declare that the research was conducted in the absence of any commercial or financial relationships that could be construed as a potential conflict of interest.

